# Recurrent skin and soft tissue infections in HIV-infected patients during a 5-year period: incidence and risk factors in a retrospective cohort study

**DOI:** 10.1186/s12879-015-1216-1

**Published:** 2015-10-26

**Authors:** Vagish Hemmige, Moira McNulty, Ethan Silverman, Michael Z. David

**Affiliations:** Division of Infectious Diseases, Department of Medicine, Baylor College of Medicine, 6620 Main, Suite 1375, Houston, TX 77030 USA; Department of Medicine, University of Chicago, 5841 S Maryland, Chicago, IL 60637 USA; Department of Pediatrics and Department of Public Health Sciences, University of Chicago, 5841 S. Maryland Ave. MC6054, Chicago, 60637 IL USA

**Keywords:** HIV, Epidemiology, Skin and soft tissue infections, MRSA

## Abstract

**Background:**

Skin and soft tissue infections (SSTIs) are common in the era of community-associated methicillin resistant *Staphylococcus aureus* among HIV-infected patients. Recurrent infections are frequent. Risk factors for recurrence after an initial SSTI have not been well-studied.

**Methods:**

Retrospective cohort study, single center, 2005–2009. Paper and electronic medical records were reviewed by one of several physicians. Subjects with initial SSTI were followed until the time of SSTI recurrence. Standard descriptive statistics were calculated to describe the characteristics of subjects who did and did not develop a recurrent SSTI. Kaplan-Meier methods were used to estimate the risk of recurrent SSTI. A Cox regression model was developed to identify predictors of SSTI recurrence.

**Results:**

133 SSTIs occurred in 87 individuals. 85 subjects were followed after their initial SSTI, of whom 30 (35.3 %) had a recurrent SSTI in 118.3 person-years of follow-up, for an incidence of second SSTI of 253.6 SSTIs/1000 person-years (95 % CI 166.8-385.7). The 1-year Kaplan-Meier estimated risk of a second SSTI was 29.2 % (95 % CI 20.3–41.0 %), while the 3-year risk was 47.0 % (95 % CI 34.4–61.6 %). Risk factors for recurrent SSTI in a multivariable Cox regression model were non-hepatitis liver disease (HR 3.44; 95 % CI 1.02–11.5; *p* = 0.05), the presence of an intravenous catheter (HR 6.50; 95 % CI 1.47–28.7; *p* = 0.01), and a history of intravenous drug use (IVDU) (HR 2.80; 95 % CI 1.02-7.65; *p* = 0.05); African-American race was associated with decreased risk of recurrent SSTI (HR 0.12; 95 % CI 0.04-0.41; *p* < 0.01). Some evidence was present for HIV viral load ≥ 1000 copies/mL as an independent risk factor for recurrent SSTI (HR 2.21; 95 % CI 0.99-4.94; *p* = 0.05). Hemodialysis, currently taking HAART, CD4+ count, trimethoprim-sulfamethoxazole or azithromycin use, initial SSTI type, diabetes mellitus, incision and drainage of the original SSTI, or self-report of being a man who has sex with men were not associated with recurrence.

**Conclusion:**

Of HIV-infected patients with an SSTI, nearly 1/3 had a recurrent SSTI within 1 year. Risk factors for recurrent SSTI were non-hepatitis liver disease, intravenous catheter presence, a history of IVDU, and non-African-American race. Low CD4+ count was not a significant risk factor for recurrence.

## Background

In 1998, the first increase in incidence of methicillin resistant *Staphylococcus aureus* (MRSA) infections in people without previous contact with the health care system was reported in children in Chicago [1]. Community-associated (CA-MRSA) infections were subsequently identified in other groups in the United States, especially marginalized populations, and CA-MRSA quickly became the leading cause of skin and soft tissue infections (SSTIs) with community onset among incarcerated populations and among impoverished urban populations [2, 3]. The advent of the CA-MRSA era dramatically affected the epidemiology of SSTIs, with a doubling in the number of SSTIs seen at safety net hospital emergency rooms [2].

Patients with human immunodeficiency virus (HIV) infection are particularly prone to infection with CA-MRSA because they are immunocompromised and because of demographic, behavioral, and socioeconomic factors as well as frequent exposures to the health care system [4, 5]. We have recently evaluated risk factors for a first SSTI among 511 HIV-infected patients in Chicago [6]. In that study, accounting for follow-up time, chronic skin conditions such as psoriasis and lymphedema, presence of an intravenous catheter, and lack of virologic suppression were most strongly associated with initial SSTI occurrence. There was also a trend toward an increased risk of SSTI in subjects receiving health insurance through Medicaid, the governmental health insurance program for the poor and disabled, and subjects with sexually transmitted diseases (STDs). Other studies have identified different risk factors [4], though our study differed methodologically from many other studies in that we identified all SSTIs which were documented in the medical record, not just SSTIs which were culture positive for *S. aureus*. This is important because in our study we found that cultures were more likely to be sent during an SSTI episode in patients with advanced immunocompromise [6]. Therefore, relying on culture data as a criterion for detection of an SSTI in retrospective studies may lead to biased conclusions about the epidemiology of SSTIs in the HIV-infected population. Although a number of anecdotal reports have suggested that recurrent SSTIs are common among HIV-infected patients [7, 8], few studies have examined the incidence of and risk factors for recurrent SSTIs in the HIV-infected population in the current era of CA-MRSA [9–12]. Accordingly, we set out to examine the risk factors for SSTI recurrence in an HIV-infected population receiving care at an urban tertiary care center clinic in the CA-MRSA era.

## Methods

### Study design

We performed a retrospective study of 511 HIV-infected adults (>18 years of age) who received their primary HIV care at the University of Chicago Medical Center’s (UCMC) Infectious Diseases Clinic for any period of time between January 1, 2005 and December 31, 2009. A description of this cohort has previously been published elsewhere [6]. SSTI was defined based on a physician diagnosis identified in medical record review. Recurrent SSTI was defined as either an SSTI at a new site or recurrence at the original site greater than 30 days after the initial SSTI with resolution of the signs and symptoms of the original infection in the interim. Individuals eligible for this study were those in the cohort who developed an SSTI and who accrued further follow-up time in the clinic after the initial SSTI. Subjects contributed follow-up time to this study from the day after the initial SSTI until either the occurrence of a first SSTI recurrence or until censoring. The study was approved by the Institutional Review Board of the Biological Sciences Division of the University of Chicago. Requirement for informed consent was waived.

### Statistical methods

Proportions between groups were compared by the χ^2^ or Fisher’s exact test, as appropriate (i.e. if any cell in the appropriate 2 x N categorical table contained five or fewer subjects). Differences between groups of continuous outcomes were compared using the *t*-test or the Wilcoxon rank-sum test. Cox regression was used for time-to event data to calculate the hazard ratio (HR) with 95 % confidence interval (CI) associated with various patient characteristics [13]. The overall fit of potential Cox models was assessed via a plot of the Nelson-Aalen cumulative hazard against Cox-Snell residuals [14, 15]. The proportional hazards assumption was verified in potential models via the use of log-log plots, and the presence or lack of influential observations was verified by calculating DFBETAs for each potential predictor [15]. Incidence rates were calculated using cluster-adjusted Poisson regression [16]. Based on exploratory analysis, we chose to categorize viral load as a binary variable with cutoff of 1000 copies/mL. CD4+ count was similarly categorized as a binary variable with cutoff of 100 cells/mL. Patients who had greater than 365 days without a measured CD4+ count or HIV viral load (VL) were deemed to have been lost to follow-up for that period and were interval censored in the statistical analysis [17].

All analyses were performed using Stata 12 (Statacorp; College Station, TX).

## Results

### Population characteristics

Of the 511 subjects in the initial cohort, 87 individuals (17.0 %) experienced at least one SSTI, as described elsewhere [6]. Two subjects from the cohort had no follow-up time after the initial SSTI, so 85 subjects were included in the analysis reported in this study. The demographics of this cohort are shown in Table 1. The 85 subjects contributed 118.3 person-years of follow-up, with subjects censored at loss to follow-up or at the time of recurrent SSTI.

**Table 1:** Baseline characteristics of individuals who did and did not develop a recurrent SSTI during the study period

Baseline variable	No recurrent SSTI (*n* = 55)	Had recurrent SSTI (*n* = 30)	Total (*n* = 85)
Sex			
Male	30 (65.2 %)	16 (34.8 %)	46 (54.1 %)
Female	25 (64.1 %)	14 (35.9 %)	39 (45.9 %)
Sexual behavior			
Male	30 (65.2 %)	16 (34.8 %)	46
Sex with men and women	6 (75.0 %)	2 (25.0 %)	8 (17.4 %)
Sex with men	11 (64.7 %)	6 (35.3 %)	17 (36.7 %)
Sex with women	13 (61.9 %)	8 (38.1 %)	21 (45.7 %)
Female	25 (64.1 %)	14 (35.9 %)	39
Sex with women	1 (100 %)	0 (0 %)	1 (2.6 %)
Sex with men	24 (63.2 %)	14 (36.8 %)	38 (97.4 %)
Mean initial age (sd)	40.7 (1.5)	40.1 (1.9)	40.5 (1.2)
Median initial CD4 (IQR)	363 (180–553)	351 (242–542)	353 (199–552)
Initial VL at time of first SSTI			
<1000	28 (70.0 %)	12 (30.0 %)	40 (47.1 %)
1000+	27 (60.0 %)	18 (40.0 %)	45 (52.9 %)
IVDU history			
No	49 (67.1 %)	24 (32.9 %)	73 (86.9 %)
Yes	5 (45.4 %)	6 (54.6 %)	11 (13.1 %)
Inhaled drug use			
No	41 (64.1 %)	23 (35.9 %)	64 (76.2 %)
Yes	13 (65.0 %)	7 (35.0 %)	20 (23.8 %)
Methamphetamine use			
No	54 (65.1 %)	29 (35.9 %)	83 (98.8 %)
Yes	0 (0 %)	1 (100 %)	1 (1.2 %)
Incarceration			
No	47 (61.8 %)	29 (38.2 %)	76 (90.5 %)
Yes	7 (87.5 %)	1 (12.5 %)	8 (9.5 %)
Race			
African-American	52 (67.5 %)	25 (32.5 %)	77 (91.7 %)
Caucasian	2 (40.0 %)	3 (60.0 %)	5 (5.9 %)
Hispanic	1 (50.0 %)	1 (50.0 %)	2 (2.4 %)
Educational status			
Did not complete high school	5 (83.3 %)	1 (16.7 %)	6 (22.2 %)
High school graduate	1 (33.3 %)	2 (66.7 %)	3 (11.1 %)
Some college	8 (66.7 %)	4 (33.3 %)	12 (44.4 %)
College graduate	4 (66.7 %)	2 (33.3 %)	6 (22.2 %)
Employment during follow-up			
No	32 (64.0 %)	18 (36.0 %)	50 (59.5 %)
Yes	22 (64.7 %)	12 (35.3 %)	34 (40.5 %)
Chronic skin disease			
No	38 (69.1 %	17 (30.9 %)	55 (64.7 %)
Yes	17 (56.7 %)	13 (43.3 %)	30 (35.3 %)
Hepatitis C			
No	47 (64.4 %)	26 (35.6 %)	73 (85.9 %)
Yes	8 (66.7 %)	4 (33.3 %)	12 (14.1 %)
Non-hepatitis liver disease			
No	54 (67.5 %)	26 (32.5 %)	80 (94.1 %)
Yes	1 (20.0 %)	4 (80.0 %)	5 (5.9 %)
Initial SSTI type			
Abscess	28 (56.0 %	22 (44.0 %)	50 (58.8 %)
Furuncle	20 (74.1 %)	7 (25.9 %)	27 (31.8 %)
Cellulitis	7 (87.5 %)	1 (12.5 %)	8 (9.4 %)
Incision and drainage performed			
No	41 (71.9 %)	16 (28.1 %)	57 (67.1 %)
Yes	14 (50.0 %)	14 (50.0 %)	28 (32.9 %)
Initial SSTI S aureus			
No	48 (68.6 %)	22 (31.4 %)	70 (82.4 %)
Yes	7 (46.7 %)	8 (26.7 %)	15 (17.7 %)
Initial SSTI MRSA			
No	50 (67.6 %)	24 (32.4 %)	74 (87.1 %)
Yes	5 (45.5 %)	6 (54.5 %)	11 (12.9 %)
Initial SSTI Treatment			
Combination therapy	11 (55.0 %)	9 (45.0 %)	20 (23.5 %)
Beta-lactam	12 (60.0 %)	8 (40.0 %)	20 (23.5 %)
Clindamycin	16 (76.2 %)	5 (23.8 %)	21 (24.7 %)
Fluoroquinolone	4 (80.0 %)	1 (20.0 %)	5 (5.9 %)
Trimethoprim- sulfamethoxazole	5 (62.5 %)	3 (37.5 %)	8 (9.4 %)
Vancomycin	2 (100 %)	0 (0 %)	2 (2.3 %)
None	3 (100 %)	0 (0 %)	3 (3.5 %)
Unknown	2 (33.3 %)	4 (66.7 %)	6 (7.1 %)
Chlamydia since 2004			
No	52 (64.2 %)	29 (35.8 %)	81 (95.3 %)
Yes	3 (75.0 %)	1 (25.0 %)	4 (4.7 %)
Gonorrhea since 2004			
No	54 (65.1 %)	29 (34.9 %)	83 (97.7 %)
Yes	1 (50.0 %)	1 (50.0 %)	2 (2.3 %)
Syphilis since 2004			
No	50 (63.3 %)	29 (36.7 %)	79 (92.9 %)
Yes	5 (83.3 %)	1 (16.7 %)	6 (7.1 %)
Human papilloma virus (Clinical) since 2004			
No	47 (65.3 %)	25 (34.7 %)	72 (84.7 %)
Yes	8 (61.5 %)	5 (38.5 %)	13 (15.3 %)
Human papilloma virus (Pap) since 2004			
No	45 (64.3 %)	25 (35.7 %)	70 (82.4 %)
Yes	10 (66.7 %)	5 (33.3 %)	15 (27.6 %)
Trichomonas since 2004			
No	47 (61.8 %)	29 (38.2 %)	76 (89.4 %)
Yes	8 (88.9 %	1 (11.1 %)	9 (10.6 %)
Any sexually transmitted disease since 2004			
No	29 (59.2 %)	20 (40.8 %)	49 (57.7 %)
Yes	26 (72.2 %)	10 (27.8 %)	36 (42.4 %)
Pneumocystic jirovecii since 2004			
No	54 (65.8 %)	28 (34.2 %)	82 (96.5 %)
Yes	1 (33.3 %)	2 (66.7 %)	3 (3.5 %)
Non-tuberculous mycobacteria since 2004			
No	53 (63.9 %)	30 (36.1 %)	83 (97.7 %)
Yes	2 (100.0 %)	0 (0.0 %)	2 (2.4 %)
Cytomegalovirus since 2004			
No	54 (64.3 %)	30 (35.7 %)	84 (98.8 %)
Yes	1 (100.0 %)	0 (0.0 %)	1 (1.2 %)
Cryptococcus since 2004			
No	53 (64.6 %)	29 (35.4 %)	82 (96.5 %)
Yes	2 (66.7 %)	1 (33.3 %)	3 (3.5 %)
Initial TMP-SMX use			
No	41 (65.1 %)	22 (34.9 %)	63 (74.1 %)
Yes	14 (63.6 %)	8 (36.4 %)	22 (25.9 %)
Initial azithromycin use			
No	48 (63.2 %)	28 (36.8 %)	76 (89.4 %)
Yes	7 (77.8 %)	2 (22.2 %)	9 (10.6 %)
Initial HAART use			
No	13 (52.0 %)	12 (48.0 %)	25 (29.4 %)
Yes	42 (70.0 %)	18 (30.0 %)	60 (70.6 %)
Initial catheter presence			
No	54 (65.9 %)	28 (34.1 %)	82 (96.5 %)
Yes	1 (33.3 %)	2 (66.7 %)	3 (3.5 %)
Initial insurance Medicaid			
No	16 (64.0 %)	9 (36.0 %)	25 (29.4 %)
Yes	39 (65.0 %)	21 (35.0 %)	60 (70.6 %)
Initial cancer status			
No	50 (63.3 %)	29 (36.7 %)	79 (92.9 %)
Yes	5 (83.3 %)	1 (16.7 %)	6 (7.1 %)
Initial diabetes			
No	48 (65.8 %)	25 (34.3 %)	73 (85.9 %)
Yes	7 (58.3 %)	5 (41.7 %)	12 (14.1 %)
Initial ESRD status			
No	48 (65.8 %)	25 (34.3 %)	73 (85.9 %)
Yes	7 (58.3 %)	5 (41.7 %)	12 (14.1 %)

Of the initial SSTIs, 50 (58.8 %) were abscesses, 27 (31.8 %) were furuncles, and 8 (9.4 %) were cellulitides. Incision and drainage of the initial SSTI was performed in 28 cases (32.9 %). Only 23/85 initial SSTIs were associated with culture data, one of which was culture-negative. *S. aureus* was an identified pathogen in 15 initial SSTIs, and MRSA in 11 of these. *Pseudomonas aeruginosa* was the only other pathogen identified in more than one patient, having been identified in 3 SSTIs. All cultures were from an infected skin site.

Of the 85 initial SSTIs, 20 (23.5 %) were treated with combination antimicrobial drug therapy. The most common combination used was ciprofloxacin and clindamycin, used in 3 patients (3.5 % of all patients, 15 % of patients receiving combination therapy). Of the 65 who did not receive combination antimicrobial drug therapy, 20 (23.5 % of all initial SSTIs) received a beta-lactam, 21 (24.7 %) received clindamycin, 8 (9.4 %) received trimethoprim-sulfamethoxazole, 5 (5.9 %) received a fluoroquinolone, 2 (2.4 %) received vancomycin, and 3 (3.5 %) received no antimicrobial drug therapy. Whether or not antimicrobial drug therapy was prescribed for the initial SSTI was not documented in the medical records of six (7.1 %) patients. As documented in the medical record, no subject underwent attempts at decolonization after the initial SSTI.

**Fig. 1:**
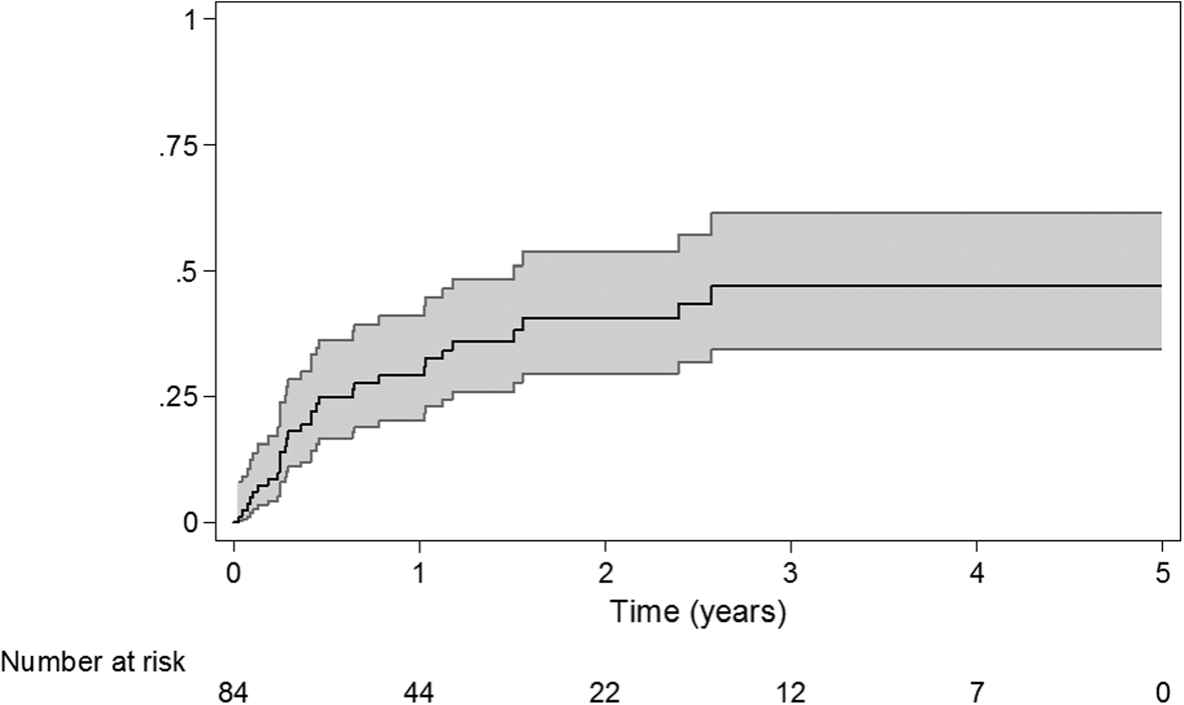
Kaplan-Meier curve demonstrating the cumulative risk of recurrent SSTI over time with 95 % confidence intervals

A total of 30 subjects among the 85 who had follow-up (35.3 %) experienced at least one recurrent SSTI during follow-up. Using Kaplan-Meier methods, the estimated proportion of individuals who would have experienced a recurrent SSTI if all individuals had been followed for one year was 29.2 % (95 % CI 20.3 %-41.0 %), while the 3-year risk was 47.0 % (95 % CI 34.4 %-61.6 %). Figure 1 shows the estimated Kaplan-Meier cumulative incidence of SSTIs over time. The incidence of second SSTI was 253.6 SSTIs/1000 person-years (95 % CI 166.8-385.7). Fourteen subjects experienced multiple recurrences (16.5 % of all subjects with initial SSTI; 46.7 % of subjects with at least one recurrence).

A number of variables assessed as potential risk factors for SSTIs were time-varying. These variables were therefore not assumed to be present throughout the follow-up period; instead, they were calculated as present or absent on each day of follow-up. For example, 79.8 % of follow-up time accrued to individuals on Medicaid, 1.6 % to individuals with a catheter, 24.7 % to individuals taking trimethoprim-sulfamethoxazole prophylaxis, and 12.0 % to individuals taking azithromycin prophylaxis. Highly active antiretroviral therapy (HAART) was being given 77.1 % of the time, and the cohort had an HIV VL < 1000 copies/mL on 60.0 % of days. The diagnosis of certain comorbid conditions was assessed similarly, with 14.6 % of follow-up time occurring in subjects with diabetes, and 7.8 % with cancer (Table 2).

**Table 2:** Proportion of patient-days with exposure to time-varying covariates

Time-varying exposure	Percentage of days of follow-up with exposure
Trimethoprim-sulfamethoxazole	24.7 %
Azithromycin	12.0 %
HAART	77.1 %
Catheter	1.6 %
Public Aid	79.8 %
Viral load	
<1000 copies/mL	60.0 %
1000+ copes/mL	40.0 %
CD4	
<50 cells/mL	6.5 %
50–99 cells/mL	4.0 %
100+ cells/mL	89.5 %
Cancer	7.8 %
Diabetes	14.6 %
End-stage renal disease	11.5 %

### Characteristics of recurrent SSTIs

The median recurrent SSTI occurred at 144 days after the initial SSTI (interquartile range [IQR], 89–377). Trimethoprim-sulfamethoxazole prophylaxis was being taken by 5/30 subjects (16.7 %) at the time of SSTI, and 3/6 (50 %) individuals with CD4+ count < 200 cells/mm^3^ at the time of SSTI were taking trimethoprim-sulfamethoxazole. The median CD4+ count at the time of recurrent SSTI was 403.5 (IQR 239–529), and the median VL was 1475 copies/mL (IQR undetectable-27800). We found that 26/30 (86.7 %) SSTI recurrences occurred in patients with CD4+ count > 100 cells/mm^3^. Of the recurrent infections, 17 were abscesses (56.7 %), 9 were cellulitis (30.0 %), and 3 were furuncles (10.0 %), and the type of SSTI was undocumented in one case. 13 of 30 (43.3 %) recurrent infections met Centers for Disease Control and Prevention (CDC) criteria for a community-associated SSTI (CA-SSTI) [18]. The primary reason that an SSTI was not classified as a CA-SSTI was hospitalization within the prior year, which was the case in 12/30 (40.0 %) patients.

Culture data were only obtained for 8 of the recurrent SSTIs, and of those, 6 of the 8 (75 %) were confirmed *S. aureus* infections, with 4 of the 6 (67 %) being MRSA. Of those 4 infections, 3 were clindamycin-susceptible MRSA, and all four were susceptible to trimethoprim-sulfamethoxazole. Of the 6 culture-proven *S. aureus* infections, only 1 MRSA SSTI met CDC criteria for being a CA-SSTI. The other two SSTIs were caused by Proteus and a coagulase negative staphylococcus species. Three of the six recurrent SSTIs caused by *S. aureus* were associated with culture data for the initial SSTI; all three initial SSTIs were caused by *S. aureus* as well. In two patients, both infections were causes by MRSA, and in one patient, both infections were causes by MSSA.

Therapy in cases of recurrent SSTI was with a beta-lactam in five cases (16.7 %), combination therapy in seven cases (23.3 %), clindamycin in four cases (13.3 %), TMP-SMX in three cases (10 %), levofloxacin in one case (3.3 %), and vancomycin in one case (3.3 %). Five patients (16.7 %) received no systemic antibacterial therapy, and antibiotic therapy was not documented in four cases (13.3 %).

### Predictors of Recurrent SSTI

The results of univariate Cox regression with possible predictors of SSTI are listed in Table 3. At any given time point, the presence of an intravascular catheter (HR 4.62; 95 % confidence intervals [CI] 1.08-19.7; *p* = 0.04), chronic liver disease (HR 2.89; 95 % CI 1.00-8.35, *p* = 0.05), and lymphedema (HR 4.12; 95 % CI 1.24-14.1; *p* = 0.02) were associated with an increased risk of recurrent SSTI, while African-American race was associated with decreased risk of recurrent SSTI (HR 0.34; 95 % CI 0.11-1.00; *p* = 0.05).

**Table 3:** Predictors of risk of recurrent SSTI in univariate and multivariate Cox regression models

Factor	Univariate HR	*p*-value	Multivariate HR (Final model)	*p*-value
	(95 % CI)		(95 % CI)	
HAART	0.67 (0.31–1.44)	0.30		
Trimethoprim-sulfamethoxazole	0.73 (0.28–1.91)	0.52		
Azithromycin	1.38 (0.48–3.98)	0.55		
Age (cont)	0.99 (0.96–1.03)	0.71		
STD (any)	1.06 (0.49–2.27)	0.88		
Chlamydia	1.18 (0.16–8.77)	0.87		
Gonorrhea	4.96 (0.65–37.7)	0.12		
Syphilis	0.49 (0.07–3.59)	0.48		
Human papilloma virus (clinical)	1.58 (0.60–4.17)	0.35		
Human papilloma virus (Pap)	1.12 (0.43–2.92)	0.82		
Trichomonas	0.45 (0.06–3.33)	0.44		
Catheter	4.62 (1.08–19.7)	0.04	6.50 (1.47–28.7)	0.01
Medicaid	0.64 (0.30–1.37)	0.25		
End stage renal disease	0.70 (0.16–2.93)	0.62		
Cancer	0.51 (0.07–3.73)	0.51		
Diabetes	1.03 (0.39–2.69)	0.96		
Hepatitis B	1.27 (0.38–4.20)	0.69		
Hepatitis C	0.91 (0.32–2.61)	0.86		
Incarceration	0.22 (0.03–1.65)	0.14		
Other chronic liver disease	2.89 (1.00–8.35)	0.05	3.43 (1.02-11.5)	0.05
Men who have sex with men	0.81 (0.36–1.81)	0.60		
IVDU	2.41 (0.98–5.91)	0.06	2.80 (1.02-7.65)	0.05
Male	0.87 (0.42–1.79)	0.71		
African-American race	0.34 (0.11–1.00)	0.05	0.12 (0.03-0.41)	<0.01
Chronic skin disease: Any	1.36 (0.66–2.79)	0.41		
Psoriasis	0.46 (0.06–3.39)	0.45		
Lymphedema	4.12 (1.24–14.1)	0.02		
CD4				
100+	Reference			
<100	1.13 (0.39–3.24)	0.83		
Viral load				
<1000	Reference		Reference	
1000+	1.58	0.22	2.21 (0.99–4.94)	0.05
Initial SSTI type				
Abscess	Reference			
Furuncle	0.26 (0.03–1.91)	0.19		
Cellulitis	0.66 (0.28–1.55)	0.34		
Incision and drainage performed on initial SSTI	1.69 (0.82–3.47)	0.15		
Initial SSTI *S. aureus*	2.10 (0.93–4.72)	0.07		
Initial SSTI MRSA	2.21 (0.90–5.40)	0.08		

Predictors with *p* < 0.2 were candidates for the final multivariate Cox model, which was developed by a forward selection algorithm. Significant predictors as well as theoretical concerns led to the choice of our final model for multivariate regression. For example, we found a strong association between lymphedema and intravenous drug use (IVDU) in our cohort; we accordingly only included IVDU in the final model to avoid collinearity. Similarly, although gonorrhea was a strong statistical predictor of recurrent SSTI in several preliminary multivariate models, we found that models including gonorrhea were overly influenced by the single subject with a history of gonorrhea during follow up with recurrent SSTI (out of two subjects with a history of gonorrhea during follow up). Accordingly, gonorrhea was not included in the final model as it was felt that the data were inadequate to allow for a multivariable analysis including this predictor. Risk factors for recurrent SSTI in the final multivariable Cox regression model were non-hepatitis liver disease (HR 3.44; 95 % CI 1.02-11.5; *p* = 0.05), the presence of an intravenous catheter (HR 6.50; 95 % CI 1.47-28.7; *p* = 0.01), and a history of intravenous drug use (HR 2.80; 95 % CI 1.02-7.65; *p* = 0.05); African-American race remained associated with a decreased risk (HR 0.17; 95 % CI 0.05-0.54; *p* < 0.01). A trend was present for HIV viral load ≥1000 copies/mL as an independent risk factor for recurrent SSTI (HR 2.21; 95 % CI 0.99-4.94; *p* = 0.05), which was nearly statistically significant. A Cox-Snell residual plot demonstrated that the model fit the data well (plot not shown).

As a sensitivity analysis, we repeated the above analyses but used 30-, 60-, 90- and 365-day recent averages for a number of the above time-varying factors, without significant change in the results (data not shown). Similarly, we used linearly extrapolated CD4+ counts and HIV VLs and found that point estimates did not change significantly. As a final sensitivity analysis, we used CD4+ count of 200 as an alternative cutoff, without significant change in the results.

## Discussion

In an urban, primarily African-American cohort, we confirmed that patients infected with HIV who had a medically attended SSTI had a high rate of subsequent SSTI, with most recurrences diagnosed within months of the initial SSTI. We identified risk factors for recurrence which do not entirely overlap the risk factors for initial SSTI found in our previous paper [19] or in the work of others [20], though our studies differed from most other studies in examining all subjects with SSTIs, not just those with proven MRSA SSTIs. It may be that the risk factors for initial SSTI occurrence differ from those that predispose to continued colonization with or re-exposure to pathogenic bacteria.

In our cohort, African Americans, who constituted more than 90 % of the patient sample, demonstrated a significantly lower rate of recurrent SSTI as compared with others. We surmise that the risk factors leading to initial SSTIs in those not of African-American descent in our cohort may be more strongly associated with the risk of SSTI recurrence than the risk factors leading to initial SSTI in African Americans. However, our sample of non-African Americans is too small to permit rigorous testing of this hypothesis. Previous observational cohorts have noted that African-Americans are less likely to remain in care [21], but differential rates of follow-up for African-Americans would only explain our results if African Americans having recurrent SSTIs were more likely than other African Americans to be lost to follow-up. We also found that established risk factors for SSTI recurrence such as intravenous drug use and presence of an intravenous catheter [8] predicted SSTI recurrence.

Some of our preliminary modelling suggested an independent impact of a gonorrhea diagnosis, although the STD incidence observed in our population was too low to assess reliably the effect of STD diagnosis on SSTI recurrence risk. It has been proposed that ongoing high risk sexual behavior may re-expose HIV-infected patients to pathogenic strains of MRSA. Prior studies by other researchers have found that many HIV-infected patients only demonstrate groin colonization with MRSA without colonization at other sites [22]. The occurrence of gonorrhea or other STDs may serve as a marker of continuing exposure to other colonized individuals, leading to reacquisition of pathogenic strains and predisposing towards SSTI recurrence.

We found that non-hepatitis liver disease, which in our cohort was predominantly due to alcoholic liver disease, was a risk factor for recurrent SSTI. Although not previously described specifically as a risk factor for recurrent SSTI in HIV-infected populations, the association between cirrhosis and SSTI risk has been previously described by other authors [23].

We were unable to test the finding of others that treatment of the initial SSTI with minocycline reduces the risk of recurrence [11] because only one patient in our study received minocycline as the treatment for the initial SSTI, which reflects institutional preferences for certain antimicrobials for the treatment of SSTIs at our center.

Our finding that lack of virologic control, better than CD4+ lymphocyte count per se, predicted SSTI risk is difficult to explain but is consistent with the findings of other authors [10]. This effect may be related to the immunologic effect of uncontrolled HIV replication. Alternatively, unsuppressed viral load, a marker of poor adherence to medication, may serve as a marker of patients who are not taking trimethoprim-sulfamethoxazole prophylaxis to prevent pneumocystis pneumonia, which potentially may also be protective against SSTI [24, 25]. Alternatively, lack of virologic suppression may be a marker of other behavioral and demographic risk factors for SSTI.

Our study is one of only six in which authors have examined SSTI recurrence in HIV-infected adults (Table 4). Skiest et al. performed a single-center prospective study at an urban, public clinic in Dallas, accruing patients with initial *S. aureus* skin infection seeking outpatient care in 2003–2004 [12]. Recurrent SSTI was noted in 11/37 patients (29.7 %). Adjustment for variable length of follow-up after the initial SSTI was not performed. Whether or not appropriate antimicrobial therapy was prescribed and whether or not incision and drainage was performed did not affect recurrence risk. No additional statistical analysis of risk factors for recurrence was reported.

**Table 4:** Summary of studies examining the incidence of and risk factors for recurrent SSTI in HIV-infected adults

Study	Population	Methodology	Outcome	Recurrence rate	Risk factors for recurrence
Crum-Cianflone et al. [10]	31 subjects with initial MRSA SSTI	Retrospective, single center	Any recurrent SSTI	41 % (all SSTI)	Lower CD4 count^a^
				21 % (MRSA SSTI)	Higher viral load^ab^
					Lack of incision and drainage^ab^
					Positive nare culture^a^
Crum-Cianflone et al. [26]	379 subjects with initial SSTI	Prospective, multi-center	Any recurrent SSTI	31 %	No significant risk factor identified
					Trend toward viral load and dermatologic condition predicting SSTI recurrence
Graber et al. [9]	62 subjects with initial MRSA SSTI	Retrospective, single center	Any recurrent SSTI	71 %	No significant risk factor identified
Vyas et al. [11]	63 individuals with initial MRSA SSTI	Retrospective, single center	Recurrent MRSA SSTI	27 %	Older age^a^
					Lower CD4 count at initial SSTI^ab^
					Lower nadir CD4 count^a^
					Higher peak viral load^a^
					Prior AIDS defining illness^a^
					History of malignancy^a^
					IVDU^*^
					Use of antibiotic other than minocycline^ab^
					Hospital admission^ab^
					Surgery^a^
Skiest et al. [12]	41 subjects with initial MRSA SSTI	Prospective, single center	Any recurrent SSTI	29.7 %	No significant risk factor identified
Present study	85 subjects with initial SSTI	Retrospective, single center	Any recurrent SSTI	35 % (raw)	Catheter^ab^
					Non-viral liver disease^ab^
					Non-African American race^ab^
					Lymphedema^a^
					IVDU^b^

^a^Univariate analysis

^b^Multivariate analysis

Graber et al. performed a single-center retrospective study at an urban, public clinic in San Francisco, accruing patients with initial MRSA infection seeking outpatient care in 2002–2006 [9]. Patients were followed through 2007. A recurrent SSTI occurred in 44/62 patients (71.0 %). Adjustment for variable length of follow-up after the initial SSTI was not performed. The median time to recurrence was 135.5 days, which was similar to the finding in our study. Cox regression performed on demographic and clinical predictors did not yield a significant predictor of SSTI recurrence. Of 62 patients with an initial MRSA SSTI, 17 (27 %) developed a recurrence.

Vyas et al. performed a single-center retrospective study at a military-affiliated HIV clinic in San Diego, accruing patients with initial MRSA SSTI in 2003–2010 [7, 11]. Patients were followed through 2010. Of 63 patients with initial MRSA SSTI, 17 (27 %) experienced a recurrent MRSA SSTI. Adjustment for variable length of follow-up after the initial SSTI was not performed. The lower rate of recurrence in this study may have resulted from the inclusion of only culture-proven recurrences. Incision and drainage was not associated with a decreased risk of recurrence. Use of an antibiotic other than minocycline and hospitalization for the initial SSTI were significant risk factors for recurrence in a multivariable analysis (Table 4). Patients with a recurrent SSTI demonstrated a significantly lower CD4+ count in a mixed-effects model using CD4+ lymphocyte count as the outcome.

Crum-Cianflone et al. performed a single-center retrospective study at a military-affiliated HIV clinic in San Diego, accruing patients with initial MRSA SSTI in 2000–2007 [10]. This study was performed at the same center as Vyas’s study in an overlapping time frame. Of 31 patients with initial MRSA SSTI, 14 (41 %) had a recurrent SSTI. The proportion of recurrence in this study differed from that observed in the Vyas study as the outcome included all SSTIs, not just SSTIs proven to be caused by MRSA. Median time to recurrence was 4 months, comparable to the results of our study. In a multivariable logistic regression model, only HIV VL > 1000 copies/mL was a significant predictor of recurrence.

Crum-Cianflone et al. examined data from the US Military HIV Natural History Study, a multicenter prospective cohort study, from 2006 to 2010 [26]. Of 379 subjects with an initial SSTI, 31 % experienced a recurrence at a median 7 months after the initial SSTI. They did not identify a statistically significant risk factor for recurrent SSTI. However, there was some suggestion in the data that the presence of a dermatological condition at the time of diagnosis of the initial SSTI (HR 1.99; 95 % CI 0.95–4.16; *p* = 0.07) and higher HIV RNA level (HR 1.18 per 10-fold increase in viral load; 95 % CI 0.98–1.43; *p* = 0.08) may have been associated with recurrence.

Our study was limited as it was a single-center retrospective medical record review. Therefore, some demographic information was missing. The diagnosis and outcome of SSTI was based on physician documentation. Older paper records were incomplete. SSTIs diagnosed at centers other than our own were missed, and so our estimate of SSTI recurrence risk was likely a lower bound. We have no specific data on how often patients in our cohort sought care elsewhere. There are community hospitals with emergency departments but no other major tertiary care centers within five miles of our center. Cox regression requires an assumption of non-informative censoring (i.e., that subjects who are lost to follow-up are similar in their characteristics to those who remain engaged in care), and the results of our analysis may be biased to the extent that this assumption is violated. However, the problem of loss to follow-up is inherent to any study of urban HIV-infected populations [27].

## Conclusions

This study contributes to the literature as one of the few analyses of risk factors for recurrence of SSTI in an urban, socioeconomically diverse cohort of HIV-infected adults. Our study is unique in that most previous studies on this topic have only included subjects whose initial SSTIs were culture-proven *S. aureus* SSTIs, while our study includes all subjects in our cohort with an initial SSTI. Our investigation improves on other studies by explicitly accounting for time-varying predictor variables and for variable time of follow-up using Kaplan-Meier methods and by following patients for a relatively long period of time. Further work to define risk factors for SSTI recurrence will enable physicians to target potential preventative interventions to those HIV-infected individuals at highest risk of SSTI recurrence.

## Abbreviations

MRSA: Methicillin-resistant *Staphylococcus aureus*
CA-MRSA: Community-associated methicillin-resistant *Staphylococcus aureus*
SSTI: Skin and soft tissue infection
STD: Sexually transmitted disease
HIV: Human immunodeficiency virus
VL: Viral load
HR: Hazard ratio
CI: Confidence interval
IVDU: Intravenous drug use
HAART: Highly active antiretroviral therapy
OI: Opportunistic infection
IQR: Interquartile range
CA-SSTI: Community-associated skin and soft tissue infection
